# Select Topics of Modern Surgery

**Published:** 1880-02

**Authors:** 


### SELECT TOPICS OF MODERN SURGERY.

1. *The Radical Operation for Non-Strangulated Hernia.* Illustrated by a case in the practice of Dr. Lee.

The history of surgery gives evidence that from the earliest times the radical cure of hernia has been a prime desideratum. As a result, a number of operations have been proposed for its accomplishment. It has been the common fate of all these operations—the ingenious ones of Gerdy, Wutzer and Rottmund not excepted—to be abandoned by the profession. The reason of this has been partly the danger of peritonitis, partly the uncertainty and instability of the results.

In trying to cure a hernia radically, we must accomplish two objects—first, the obliteration of the sac of the hernia; and second, the obliteration (umbilical, ventral, crural hernia) or sufficient diminution in width (inguinal hernia) of the ring (crural, umbilical, external inguinal) of the hernia—*i. e.*, the opening in the fibrous tissues of the wall of the abdomen. The older methods of obliteration of the sac, as actual cautery, castration, excision with or without ligature of the neck of the sac, introduction of foreign bodies into the sac with a view to cause adhesive inflammation, etc., were intended to effect radical cure themselves; the narrowing of the ring was left to nature. Most of the patients so operated upon died in consequence, while many of the survivors had early return of the hernia through the open ring and by the side of the obliterated sac. Thus professional attention was directed strongly to the importance of the ring of the hernia, at the beginning of the present century. The obliteration or plugging of the ring was sought to be effected by transplantation of a flap of skin (one successful case, Jameson), or through invagination of the skin of the scrotum (Gerdy, Wutzer, Rothmund, Wells, etc). The latter method has been practiced extensively. Although relatively bloodless, it is not without danger of peritonitis, and the plugging of the ring is very imperfect and uncertain.

A more rational way of obliterating the columns of the fibrous ring was, in 1863, introduced by Wood, of London. He unites the columns of the crural ring with sutures, introduced subcutaneously through a small incision in the scrotum. The uncertainty of carrying the sutures into the columns, guided only by the feeling of the fingers, is obvious, and probably this is the reason why relatively few surgeons have adopted this method.

It is to Lister's antiseptic method of operating, and to his antiseptic dressings, that we are indebted for the last important step toward effective radical cure for non-constricted hernia.

Czerny, the most gifted of Billroth's pupils, has, in a daring but strictly scientific way, proposed and successfully employed a new method, the one used in the operation upon the patient, whose case will be presently described.

The main features of the operation are: a free incision over the whole length of the hernia; exposure of the sac; reduction of the contents of this, if possible, without opening it, but with opening if this be necessary; ligature of the neck of the sac and amputation of its fundus; reduction of the ligatured stump of the sac into the abdomen, the fundus being removed or allowed to remain in its place. Next the fibrous columns of the ring are dissected out and a suture of strong catgut is passed through them, bringing them together; after which the external wound is closed over drainage tubes and Lister's dressing applied.

Previous methods were applicable only to reducible hernias, and could not be used without great danger, if even the smallest portion of the contents was adherent to the sac.

In the case now to be related, the operation was made upon an irreducible hernia. The patient, E. C., thirty-one years old, a switchman, noticed, in 1864, a small inguinal hernia on the right side; it was of the size of a large marble. It only came out occasionally with efforts at jumping or other violent straining effort, and he was obliged always to replace it manually. It caused no pain. In the course of years it grew to the size of a duck's egg, but he could always return it easily.

On June 7th, 1879, while forcing over a ground switch with great effort, the hernia came down much larger than ever before, forming a tumor in the scrotum seven inches long by three and a half or four inches broad. He went home and to bed, tried to reduce the hernia and succeeded partially, but a considerable mass remained protruding and in the scrotum. The next morning there was some pain in the tumor, and a physician attempted to reduce it, with the patient under anæsthesia, but in vain. Two days later, Dr. Lee was called, and drew a little serous fluid from the tumor with the aspirator, and then attempted to reduce, but without success. There remained an irreducible hernia, four inches long by three inches wide. A few days afterward a careful examination was made, when the tumor was found of the same size as before, a little tender on pressure, giving a dull percussion sound over its whole surface; the upper part of the tumor movable from side to side, the upper, outer, narrower part fixed to and filling the external inguinal ring (?), no fluctuation being discoverable anywhere. The contents of the tumor were felt to be an uneven, somewhat nodular mass, over which the integuments, skin, dartos and fastia were movable. There had never been any hiccough, vomiting or constipation, or other evidence of strangulation of gut or even irritation of peritoneum or contents of the peritoneal cavity.

The diagnosis arrived at was irreducible inguinal hernia omentalis (epiplocele). The patient, unable to do his work and earn his living, begged that something might be done to relieve him. As no bandage or truss would enable him to do the heavy work required by his vocation, it was explained to him that the only means by which he could regain his health and former ability to work was an operation for the radical cure of hernia. The dangers and chances were laid before him, and he consented to the operation. Five days previous to the operation he was put to bed, given a dose of castor oil every day, and kept on a diet of liquid food, consisting of beef tea and milk in moderate quantity.

On the 30th of July, at 3 P.M., the operation was performed by Dr. Lee at the patient's home. There were present Drs. Fenger, Isham, Bridge, Saulsbury and Goldspohn.

The pubis had been shaved and the abdomen washed carefully with soap and carbolized water.

The anæsthesia was induced with ether, and every step of the operation was made under a powerful spray of five per cent. carbolic acid solution.

An incision six inches long was made from above the neck of the hernia and the external ring down to the scrotum. The superficial fascia, tunica, dartos and the fascia of the external oblique were cut through with only some venous hemorrhage. The sac of the hernia, thus laid bare, was dissected loose from the surrounding tissue and opened, when about an ounce of reddish, but clear, serous fluid escaped. From this moment, and during the rest of the operation in the sac, the neck of the latter was firmly compressed with the fingers to prevent blood or other fluid from escaping into the abdominal cavity. Through the dilated opening the sac was found to contain a large quantity of somewhat indurated and œdematous omentum, but no intestine. The omentm was then divided at the neck of the hernia into three equal parts by means of ligatures, no knife being used, and separately ligatured firmly, carbolized catgut being the material used. The mass was then cut off one centimeter from the ligatures, and the stump pushed through the ring into the abdomen. The next step was to carefully separate the empty sac from the spermatic cord, when one strong catgut ligature was passed around the neck, and the peripheral part of the sac cut off.

The fibrous columns of the external and inguinal ring were next laid bare, the opening being about four centimeters long by one and a half centimeters broad, and, by means of two curved needles threaded with *one* strong, large and long piece of catgut, a continuous, uninterrupted suture, resembling the lacing of a shoe, was passed through the columns 0.5 centimeters from their free border. The needles were always passed from within outwards, and alternated from side to side, so that the catguts crossed each other as in the lacing of a shoe. In this way the external inguinal ring was closed, the columns being drawn together firmly, a space being left at the internal inferior point of the ring just sufficient to accommodate the spermatic cord without strangulation. After hemorrhage had ceased, a large drainage tube was laid in the bed of the wound, and the integument closed over it with carbolized silk ligatures.

A perfect Lister antiseptic dressing was applied over the whole hypogastric region, with an opening for the penis. The dressing was fastened with a double spica, and the whole bandage covered with a large, elastic, pure rubber bandage, with a view to excercise an uniform elastic pressure over the wound as well as over the whole lower part of the abdomen.

The patient was put to bed, with a pillow under the knees, to maintain them in a semi-flexed position. Laudanum was given to prevent the bowels from moving.

On August 1st the wound was re-dressed. There was no fever and no pain in the abdomen.

August 5th the dressings were re-applied. The bowels had moved for the first time since the operation. There was a very litle discharge into the dressings.

On August 7th the patient had a slight chill in the morning. The pulse was 100; temperature ($101\frac{1}{2}$ F.), 38.7°. There was some pain in the middle of the wound, and a hard swelling could be felt between the latter and the radix penis, about three centimeters below the external inguinal ring. The lower half of the drainage tube was taken out.

August 9th there was found to be but little discharge in the dressings; fever had been absent twenty-four hours; the swelling mentioned had increased in size and was steadily painful. During the night the pain suddenly ceased, and when the next day (August 10) the dressings were removed, they were found to be considerably infiltrated with pus; pus was issuing from the canal of the drainage tube; the swelling had considerably decreased in size, and pressure upon it caused pus to well up from the canal of the tube.

August 12th, two small pieces of substance, resembling in size and appearance rice grains, came from the wound.

By the 17th of August the little swelling had disappeared; there was some discharge through the drainage tube, and the patient felt well.

On the 26th day of August, the discharge having nearly ceased, the upper and last half of the drainage tube was removed.

By September 1st the wound was nearly healed, and the patient was allowed to get up.

On September 9th he went to work, it being the forty-first day after the operation. The wound was entirely healed.

He wore a light truss for a month after going to work, but it caused a slight excoriation of the epithelial covering of the scar, and had to be dispensed with.

For (now) two months he has not worn any support over the scar.

This man is now able, and has been, to pursue his occupation without inconvenience. He can jump upon and off cars, and perform all the movements of his body with freedom and without inconvenience.

The actual condition of the part operated upon is now as follows: there is a reddish scar ten centimeters long, reaching from the external inguinal ring to the scrotum; it is hard and firm, but not tender. When the man coughs there is no greater protrusion at this point than of the skin covering the external ring of the opposite side.

Invaginate the skin of the scrotum, and pass the finger up toward or into the external ring, let the patient cough, and there is felt *less* pressure from within than may be felt on the left side, where no hernia ever existed.

So far, therefore, as may be judged from the patient's present condition, the success of the operation—the radical cure of the hernia—is complete.

Let us now consider the three main points of this new operation, namely, the treatment of the sac, the contents of the hernia, and the external fibrous ring in the wall of the abdomen.

*(a.) The Sac of the Hernia.*—Considering the sac as a part of the peritoneal cavity, the surgeon naturally desires to avoid opening it. This may be done in small reducible hernias with non-adherent sac, it being possible to loosen the sac without opening it, and to pass it into the abdomen and to close up the fibrous ring outside of it. Czerny recommends this plan for small umbilical hernias only. We should not advise this practice in inguinal hernias for the following reasons: first, the reduced sac would be liable to pass down through the opening in the ring we are obliged to leave for the cord; second, in closing up the neck of the sac by means of sutures or ligatures, we would lose the advantage of a remedy in some cases by itself alone capable of producing radical cure of hernias, as has been seen after the operation for strangulated hernia.

Opening of the sac without exposure of its internal surface can be done by dissecting loose the neck from its surroundings, applying single or double ligature in two places, and cutting through between these two. The upper ligatural part can then be returned to the abdomen, while the lower part, the fundus, is left in its place or extirpated entirely—which plan is preferable, as will appear further on.

Opening of the sac is of course inevitable whenever the hernia is wholly or partially irreducible. The danger from exposure of the internal wall of the sac is, in our opinion, mainly if not entirely obviated by the antiseptic method of operating. The statistics accessible to us give eleven cases (Czerny five, Socin five, our case one), with no peritonitis in any of them.

The contents of the sac disposed of in the way to be more fully described later, the neck of the sac ligatured and cut off and united with sutures (one of Czerny's cases) and pushed back into the abdomen, the question is what to do with the fundus—whether to leave it or remove it.

In four cases (Czerny three, our case one), where the sac was extirpated, the recovery was four times interrupted by the formation of an abscess near to or in the upper part of the scrotum, *i. e.*, in the place of the extirpated fundus of the sac. The abscess made its appearance the fifth day and the seventh day in one case each, and on the fourth week in two cases. In one of Czerny's cases he left the fundus, washing it out with a five per cent. solution of carbolic acid, and no abscess interrupted the recovery.

Czerny is inclined to ascribe the formation of abscess to the mechanical molestation of the tissues of the spermatic cord, in dissecting it loose from the frequently very adherent walls of the sac. He therefore suspects that this inflammation can be avoided by leaving the fundus in its place. But the fundus left in this manner might fill up afterward with serous fluid and form a cyst, with all the inconvenience of a hydrocele of the cord. To avoid this, an adhesive inflammation and obliteration of the sac might be obtained by cauterizing its inner surface with equal parts of carbolic acid and alcohol, as is the practice of Shunch in the radical operation for hydrocele.

Experience must in future decide whether it is desirable to leave and cauterize or remove the fundus of the sac. Certainly an abscess should, if possible, be avoided, as it is very desirable that an uninterrupted healing by first intention should take place. Inflammation in the wound after this operation might endanger the results in a double way. The suppuration might extend up through the ring, along the ligatured neck, and cause fatal peritonitis. It would be less dangerous, but fatal enough to the success of the operation, if the suppuration around the sutures of the fibrous ring caused reopening of the latter and the discharge of the material of the former. Experience has not tended to emphasize this apprehension of danger. In all the observed (4) cases of abscess, this proved harmless to the life of the patient, as well as the final results of the operation.

(*b.*) *The Contents of the Hernia—Intestines, Omentum.*—An anse of the intestines of an irreducible hernia may be adherent to the anterior walls of the sac, and requires the utmost caution in opening the sac. The intestine in most cases contains air as well as fæces, and betrays its existence by tympanitis or want of absolute dullness on percussion. But we must bear in mind that there is always a possibility of finding, in spite of actual dullness on percussion, a knuckle of intestine close to the wall of the sac. No cut should be made without the director, and this must be used with such slight force as not to puncture an adherent gut. Never push the director through bleeding tissues, and always recognize the nature of the tissues covering the furrow of the instrument (connective tissue or muscular wall of the intestines) before cutting them through. Try to find a place where the wall of the sac can be seen to move over the surface of its contents, and where it consequently can be opened without danger.

The sac being opened the intestine must be reduced. If no adhesions, but a too narrow ring opposes the reduction, we must dilate as in strangulated hernia. Adhesions require great care and skill in detaching the intestine. They (*i. e*, adhesions) may be torn with two pairs of forceps, cut with a blunt-pointed pair of straight or curved scissors, or with the knife; but they should be cut off (if at all) close to the wall of the sac, leaving parts of the latter in connection with them if they are very short. Every bleeding part of cut off adhesion must be ligated carefully with fine catgut (in one of Czerny's cases six catgut ligatures were required), that not a drop of blood escapes from the surface of the reduced intestine.

The omentum may be reduced if it is healthy, and the reduction is easily done. Rather than dilate the neck and ring, we should ligate the pedicle of the omental mass, wholly if it is small, dividing it if voluminous, and then cut it off external to the ligatures. Catgut or carbolized silk may be used for this purpose.

(*c*) *The Fibrous Ring.*—The interrupted sutures may be used. (One of Czerny's cases.) A more efficient closure of the ring seems to be obtained by means of the uninterrupted suture (used in four of Czerny's cases and in our own case) heretofore described. As the material for this suture we must recommend strong carbolized catgut. As the catgut after some time—the length of which is as yet unknown—becomes soft and gives way in the progressing process of absorption, it would be desirable if we could use a more resisting material, namely, strong silk ligatures. We would thus be more entitled to expect the crura of the fibrous ring to remain united for a longer time, probably long enough to effect a permanent union, so as to avoid relapse of the hernia, or rather the coming out of a new one.

The well known experience from intra-abdominal operations of later years would seem to permit the use of the silk. To further the solution of this problem, Czerny made experiments with silk disinfected in various ways, testing it as to its power of producing decomposition, viz: formation of bacteria in Pasteur's fluid. His experiments seemed to prove that heavy silk thread boiled in a five per cent. solution of carbolic acid would be innocent, aseptic, and the material to use for the operation. In this way he made use of silk sutures in a case of inguinal hernia on both sides. The two operations were made at the same time on the 9th of March. On the 20th and 24th the sutures came out from the left and right sides respectively. Three months after the operation there was no hernia on the right side, but on the left a tumor as large as a pigeon's egg came out when the patient was made to cough. This one case, although not sufficient to decide the question finally, was so strong against the silk that we resorted to the catgut—tha present case—and as the question now stands we shall continue to use it.

The future must decide whether the good results of this new operation for the radical cure of hernia are permanent. The small number of cases and the short time that has elapsed since the operations leave the question undecided.

Socin had four successful cases of operations for inguinal hernia, no relapses after one year. In one unsuccessful case the hernia returned after three months, and was operated upon the second time eleven months later. For Czerney's cases from 1875, we are yet unable to find any further information as to the stability of the results. That this stability will to a great extent be dependent upon the nature of the case and the tissues of the patient is obvious. But it will scarcely be denied that, delicate and often difficult as this operation is, the results will depend upon the care and skill of the operator to a much larger extent than in many other surgical operations. We should not advise any surgeon to perform this operation who is not a believer in, and thoroughly familiar with, the antiseptic method in all its smallest and often tedious details.

But following Lister's method of operating and after treatment, we believe the radical cure of hernia is as safe or safer than the old sub-cutaneous methods, and that we are indebted to Czerny for an ingenious, scientific and practically valuable step toward the solution of the old question of the radical cure of non-strangulated ruptures.—*Chicago Med. Jour. and Exam.*

